# Correction

**DOI:** 10.1111/cas.15825

**Published:** 2023-05-23

In an article^1^ titled “The effects of cannabidiol via TRPV2 channel in chronic myeloid leukemia cells and its combination with imatinib” by Federica Maggi, Maria Beatrice Morelli, Daniele Tomassoni, Oliviero Marinelli, Cristina Aguzzi, Laura Zeppa, Massimo Nabissi, Giorgio Santoni, Consuelo Amantini, there was an error in Figure 1B.

The correct Figure 1 is shown below:

**FIGURE 1:**
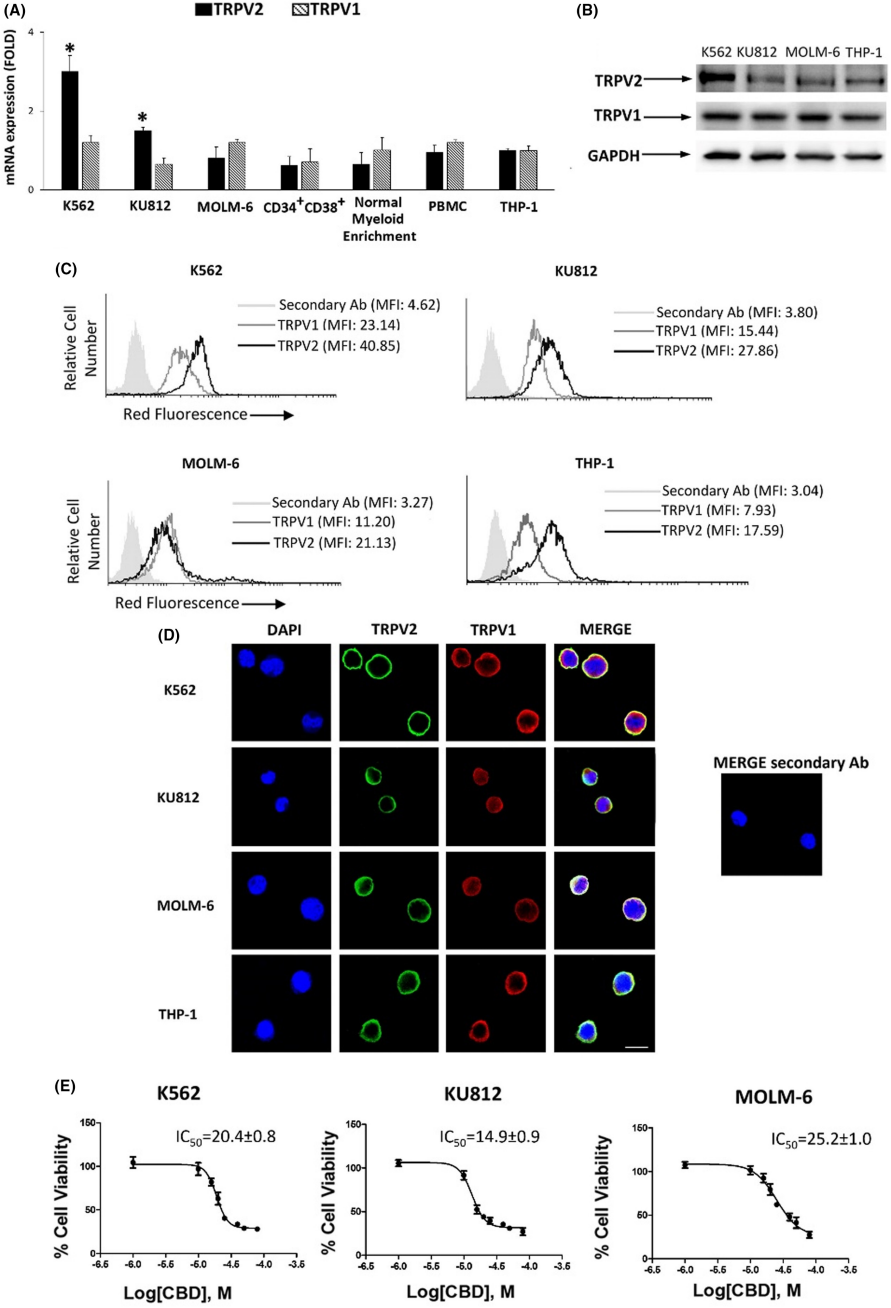


The authors apologize for the error.
